# Beyond the Grant–Stebbins model: floral adaptive landscapes and plant speciation

**DOI:** 10.1093/aob/mcaf096

**Published:** 2025-07-31

**Authors:** Kathleen M Kay, Bruce Anderson

**Affiliations:** Department of Ecology and Evolutionary Biology, University of California, Santa Cruz, Santa Cruz, CA 95060, USA; Department of Botany and Zoology, Stellenbosch University, Matieland 7602, South Africa

**Keywords:** Angiosperms, floral adaptive landscapes, flowers, Grant–Stebbins model, most effective pollinator principle, natural selection, pollination, plant speciation

## Abstract

**Background:**

Floral diversity, a striking feature of angiosperm evolution, provides the impetus and rationale for linking pollinator-driven selection to speciation processes. Perhaps the most widely adopted model for pollinator-driven speciation is the Grant–Stebbins model, in which plant populations locally adapt to the most effective pollinator, leading to floral ecotype formation and, eventually, reproductive isolation and speciation. However, modelling and empirical studies suggest that populations need not adapt to the most effective pollinator, and major floral transitions remain poorly explained.

**Scope:**

We evaluate the Grant–Stebbins model, focusing especially on the most effective pollinator principle. We use floral adaptive landscapes to articulate a more complete and accurate framework for understanding floral adaptation, starting with the premise that plants evolve to maximize fitness. We highlight ways to improve the assessment of pollinator fitness functions, both singly and in combination. We show how floral adaptive landscapes can be used to describe processes of floral adaptation within populations, evolutionary transitions between floral phenotypes, and a variety of real-world situations that do not fit neatly under the Grant–Stebbins model. Finally, we evaluate how floral adaptive landscapes can clarify the role of pollination in speciation under a variety of species concepts.

**Conclusions:**

The Grant–Stebbins model, while inspiring decades of empirical studies, is a caricature of pollinator-driven speciation and explains only a limited range of adaptive outcomes. By using adaptive landscapes, we acknowledge that flowers are not adaptations to the most effective pollinator, but adaptations to maximize fitness, making evolutionary shifts between distinct floral phenotypes easier to understand in multi-pollinator environments. Finally, we argue that while pollinators often drive floral divergence, speciation most likely results from simultaneous divergence in multiple niche axes across a geographic range, which has been underemphasized in plant speciation research.

## INTRODUCTION: THE GRANT–STEBBINS MODEL AND POLLINATION ECOTYPES

Floral divergence is one of the most striking features of angiosperm evolution and is thought to be intimately tied to speciation processes in many plant lineages. As plant populations adapt to different pollinators across a geographic landscape, speciation occurs because the populations become morphologically distinct, ecologically distinct and reproductively isolated. This process has been dubbed the Grant–Stebbins model, after the early contributions of Verne and Karen Grant and G. Ledyard Stebbins, becoming the most widely accepted conceptual basis for how pollinator-driven ecological speciation occurs (Johnson, 2006; van der Niet *et al.*, 2014). In particular, Stebbins (1970) provides a verbal model describing how plant populations adapt to different pollinator functional groups. He states that ‘the characteristics of the flower will be molded by those pollinators that visit it most frequently and effectively in the region where it is evolving’, an idea that became widely known as the most effective pollinator principle (MEPP). Moreover, he asserts that floral divergence occurs along ‘lines of least resistance’ determined by the constraints of current floral traits and the external environment. The model implies that trade-offs in the phenotypic requirements of different pollinator functional groups result in floral syndromes that impart specialization on the most effective pollinator (MEP). In other words, adaptation to the MEP is expected to reduce the effectiveness of other pollinators in a multi-pollinator environment. This work built upon two important contributions. First, Grant (1949) showed that floral characters are often used for species-level taxonomic identification, especially in plants with specialized bird, bee and long-tongued fly pollination, suggesting the importance of floral divergence in the speciation process. Second, Grant and Grant’s (1965) study of pollination in the Polemoniaceae asserted that floral ecotypic divergence occurs across a geographic range in response to what they called a geographically variable ‘pollination climate’ and that ecotypic divergence eventually contributes to reproductive isolation. Thus, pollinator-driven speciation comprises adaptation to different MEPs among plant populations, the formation of floral ecotypes, and the contribution of floral divergence to reproductive isolation. The Grant–Stebbins model has spawned extensive work identifying the most effective pollinators in plant populations (e.g. Schemske and Horvitz, 1984; Mayfield *et al*., 2001) and quantifying contributions of floral divergence to reproductive isolation among young plant taxa co-occurring in sympatry (reviewed in Johnson, 2006; Kay and Sargent, 2009; van der Niet *et al*., 2014).

Despite its influence, the simple verbal model underlying this approach has several limitations. Pollinator effectiveness was not formally defined by Stebbins, and is measured and discussed in a variety of inconsistent ways in the literature (reviewed in Primack and Silander, 1975; Spears, 1983; Inouye *et al*., 1994; Ne’eman *et al*., 2010; Schupp *et al*., 2017). Grant and Stebbins typically used ‘pollinator’ to mean pollinator functional group, but these can be broad or narrow and comprise multiple pollinator species that are not necessarily identical in effectiveness (Fenster *et al*., 2004). Perhaps more importantly, Aigner (2001) pointed out that plants should evolve to maximize fitness, not adapt to the single most effective pollinator, and he described scenarios of adaptive landscapes under which floral adaptation to pollinators may not follow the MEPP. Yet this perspective has not been widely implemented in studies of pollinator-driven selection, perhaps because of the complexity of Aigner's models or the logistical difficulties in estimating fitness contributions from a suite of visitors. Consequently, the oft-invoked MEPP fails to explain pollination systems that are not highly specialized, because plants are expected to continuously adapt to the single MEP in a community (e.g. Mayfield *et al*., 2001; Aigner, 2004; Pauw *et al*., 2020). In particular, the MEPP downplays the importance of secondary vectors in the evolutionary trajectory of flowers, suggesting that generalization is not adaptive, but simply a transient phase that plants pass through as they shift from one pollinator functional group to another. For this to occur, Stebbins (1970) envisaged an intermediate phase of ‘double function’ where both the ancestral and the novel pollinators are capable of pollinating the flower. A point of confusion resulting from the Grant–Stebbins model is that strong floral isolation requires derived floral traits that do not work well with, and even deter, ancestral pollinators, yet these traits seem the least likely to evolve by natural selection during a transition through a phase of double function. For example, it is hard to envisage how a red flower that attracts birds but deters bees evolves from a bee-attracting blue flower via an intermediate stage when both bees and birds are pollinating. Thus, the MEPP does not explain the process, timing or order of traits by which floral isolation arises. The focus on adaptation to the MEP also requires invoking large fluctuations in pollinator abundance or expansions of plant range for pollinator shifts to occur – fluctuations and expansions that may not be ecologically realistic or even necessary. Finally, a pervasive focus on pollinators as the drivers of reproductive isolation in sympatry downplays the important role of the ecogeographic isolation that accompanies most floral divergence and plant speciation. This, despite Grant’s (1949) assertion that ecogeographic and pollinator isolation act synergistically in the speciation process. Here, we embrace a more complex view of how plants maximize fitness and how floral divergence contributes to speciation. We rely on adaptive landscapes to describe processes of floral adaptation within populations, evolutionary transitions between floral phenotypes, and contributions of floral divergence to speciation processes. We illustrate key points with examples, primarily from our work in California, South Africa and the Neotropics, rather than undertaking a comprehensive review.

## HOW DO PLANTS ADAPT TO THE LOCAL POLLINATION CLIMATE?

**Fig. 1.**
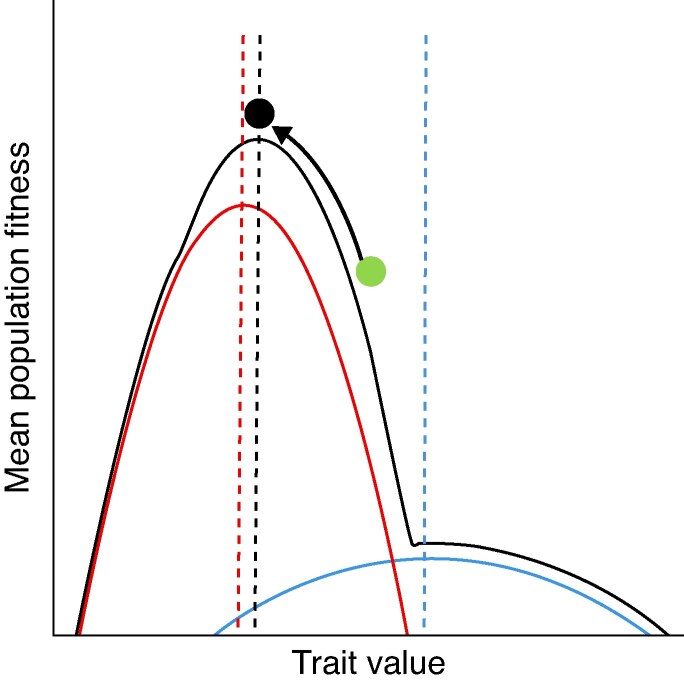
A hypothetical floral adaptive landscape adapted from Aigner (2001) showing the contributions to fitness made by two different pollinator types (solid red and blue lines) over a simplified 2-D range of trait values (e.g. corolla tube length). Each pollinator generates a contribution to total plant fitness with optimal phenotypes depicted by red and blue dashed lines, and together they generate a combined (in this case, additive) adaptive surface (solid black line) and optimal phenotype (black dashed line). The black line has been slightly offset (upwards), so that it does not obscure the other two lines. Plant populations should always evolve in a manner that increases mean population fitness, moving up adaptive slopes. For example, if the starting population's average trait value is the green dot, the plant population would be expected to evolve reductions in this trait until it reaches a point that corresponds with the black dot (optimum floral phenotype). Here, the optimal floral phenotype roughly aligns with the fitness peak generated by the most effective pollinator (red dashed line) as one may predict from MEPP; however, close inspection reveals that the combined contributions of the blue and red pollinator make the alignment imperfect. Importantly, an MEPP perspective on the population at equilibrium would ignore the contribution of the blue pollinator.

Before addressing the issue of how plant populations diverge in floral phenotype, we first focus on how pollinator-driven selection works within a population. Plants should evolve to maximize fitness, instead of adapting to the most effective pollinator (Aigner, 2001). This reframing changes the task from evaluating which pollinator is most effective in each population to studying how plant fitness varies with different combinations of floral traits and how each floral visitor contributes to the overall fitness function, hereafter ‘floral adaptive landscapes’ (Fig. 1, Box 1). Under this framework, the optimal floral phenotype in a population depends on how the fitness contributions by different pollinators combine and interact to affect total plant fitness. While floral morphology may often be closely aligned to the morphology of the most effective pollinator at a site, there are many situations where floral adaptive landscapes show selection for a different optimum. For example, floral adaptive landscapes can account for floral morphologies that appear to be adapted to less effective pollinators or very generalized floral morphologies that are highly adapted, but reflect a compromise among multiple different pollinators. Floral adaptive landscapes may also be used to understand the different and sometimes conflicting contributions made by male and female floral functions, and these also can explain floral phenotypes that do not intuitively match the morphology of what appears to be the most effective pollinator. We explore a variety of scenarios and issues to consider when shifting from the MEPP to a floral adaptive landscape perspective, giving examples to illustrate key points and identifying practical solutions to enhance our understanding of floral adaptation within populations.

Box 1 AN EXPANDED VIEW OF FITNESS IN THE CONTEXT OF FLORAL EVOLUTION

Fitness is the relative contribution of an individual to future generations and, thus, is exceedingly difficult to measure empirically. Instead, we measure components of fitness, chosen because of their predicted relationship with fitness, their close association with putative agents of selection, or their ease of measurement (reviewed in Wadgymar *et al*., 2024). Studies of pollinator-driven selection have most often quantified pollinator visitation rates, pollen deposition on stigmas, and/or seed set, and related these measures to floral phenotypes. Each of these measures has limitations, and all ignore the important contributions of siring success through pollen and variation in offspring quality. We review these issues and suggest ways forward to better quantify floral adaptive landscapes.

**Pollinator visitation rates**

Visitation rates are typically the easiest data to obtain, although they are not always related to a visitor's fitness contribution in a straightforward way because of variation in the amount and quality of pollen exported and deposited (Schemske and Horvitz, 1984; Vazquez *et al*., 2005). Visitation rates provide important information on spatial and temporal variation in floral adaptive landscapes, but, ideally, are interpreted along with other fitness measures. One relatively simple improvement is to only include visits in which the pollinator makes contact with the anthers and/or stigma.

**Pollen deposition**

On a per-visit basis, pollen deposition can vary widely (e.g. Herrera, 1987; Page *et al*., 2021) and can be more closely tied to female fitness than visitation rates. It may also provide information on heterospecific pollen transfer, which can have negative fitness repercussions (Moreira-Hernández and Muchhala, 2019). Nevertheless, pollen deposition has several limitations as a fitness component. For many reasons, pollen deposition may not scale linearly with seed set. Deposition often exceeds ovule number, and a variety of pollen–pistil and pollen–pollen interactions (e.g. self-incompatibility, pollen competition) can affect the relationship between deposition and seed set, making analysis complicated. It may also be difficult to distinguish conspecific pollen, especially in species-rich communities. A possible improvement is to harvest pistils and assess pollen tube growth in the style instead of, or in addition to, counting pollen grains on the stigma.

**Seed production**

Seed set and fruit set (two frequently correlated fitness metrics) are more closely related to fitness, at least through female function, than visitation or pollen deposition. However, they may sometimes tell us less about pollinator selection than visitation or pollen receipt because they can both be strongly affected by other selective forces, such as resource acquisition or herbivory. Testing for pollen limitation may be an important first step in assessing the usefulness of this variable. Alternatively, to isolate pollinator-driven selection when using seed set as the fitness component, one can use supplementary hand pollination of a subset of plants and subtract estimates of selection gradients for plants receiving supplementary hand pollination from estimates obtained for open-pollinated control plants (Galen, 1996; Sletvold *et al*., 2010).

**Siring success**

From studies of dioecious and monoecious plants, and from sexual selection theory, we expect pollinator-driven selection through male function to often have different optima and/or intensities compared with selection through female function. It is also clear that pollinators can differ in their per-visit effects on pollen dispersal, especially when there are differences in foraging patterns and grooming behaviour (e.g. Wilson and Thomson, 1991). Nevertheless, because of the difficulties in measuring pollen export and paternity, fitness through male function has been largely ignored in studies of pollinator-driven selection (reviewed in Austen and Weis, 2016; Minnaar *et al*., 2019*a*). Siring success through pollen export can be tracked indirectly through the use of coloured dye, naturally existing pollen morphs, or quantum dots (Thomson *et al*., 1986; Minnaar *et al*., 2019*a*). It can also be inferred by quantifying aspects of the pollen economy for different visitors, such as pollen carryover curves and relative pollen export (Thomson, 2003; Johnson and Harder, 2023). Selection through male fitness may be measured directly through genetic paternity analysis (Morgan and Conner, 2001) or by evaluating the response to selection in an experimental population for which female fitness is held constant (Austen and Weis, 2016). Each of these approaches varies in its feasibility depending on the system, but we see estimating pollinator-driven selection through male fitness as one of the areas in which the most future progress can be made in understanding floral evolution. Its importance lies in the fact that it is sometimes assumed that selection on floral traits acts more strongly through the male fitness pathway than the female fitness pathway. Although in reality, the relative importance of male versus female fitness function may simply depend on variation in ecological conditions (e.g. pollen limitation versus an excess of pollen).

**Offspring quality**

Because of differences in pollinator foraging behaviour, flight distances and pollen carryover, the quality of offspring resulting from different pollinators may vary greatly, and the contribution of an individual to future generations may not have a linear, or even positive, relationship with any of the aforementioned fitness measures. For certain life histories and environments, such as long-lived plants in relatively stable environments, fitness is less dependent on offspring number and more dependent on offspring quality, yet very few studies have tracked offspring quality in the context of pollinator-driven selection. Ideally, progeny could be followed through the next generation in ecologically realistic settings. Less labour-intensive methods could use molecular markers to estimate inbreeding coefficients of progeny produced by different pollinators (e.g. Valverde *et al*., 2019) and to estimate inbreeding depression (e.g. Ritland, 1990).

### Flowers can simultaneously adapt to multiple pollinators

**Fig. 2.**
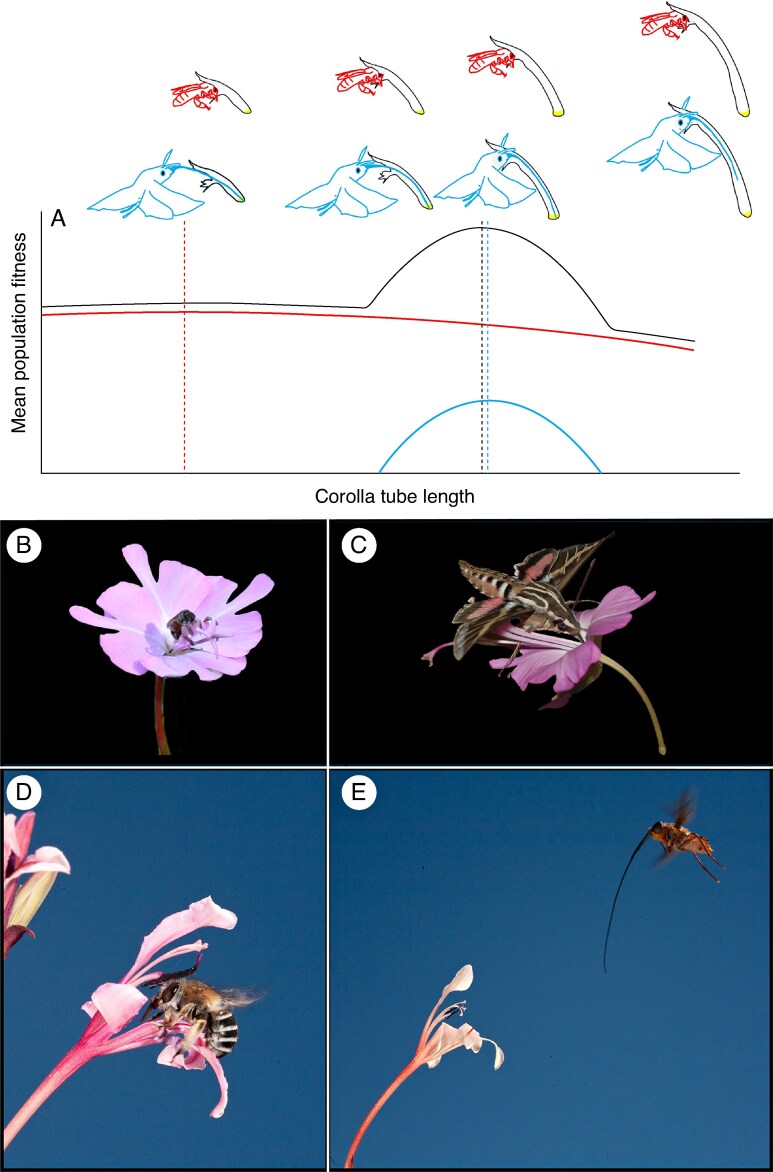
Adaptation to the pollinator with the steepest fitness function. (A) In a hypothetical example following Aigner (2001, Fig. 1C) and inspired by *Castilleja sessiliflora* (Wenzell *et al*., 2024), pollen-collecting bees (red) may visit long- and short-tubed flowers in the same way, and consequently their fitness contributions change little across a range of floral tube length. As a result, bees impose weak selection on floral tube length, even if they are very effective pollinators. In contrast, the long-tongued hawkmoth pollinator (blue) is only effective over a narrow range of trait values because its body does not make contact with short-tubed flowers (left) and it avoids flowers with inaccessible nectar (right). Because fitness changes quickly with changing trait values, selection by hawkmoths on tube length is strong, and the overall optimal floral tube length (black dashed line) broadly matches the optimal tube length predicted by hawkmoths (blue dashed line). The optimum is driven by the hawkmoth regardless of whether it is the MEP or the least effective pollinator, as shown here. (B, C) In another example, *Clarkia breweri* (Onagraceae) flowers have several quintessential traits of a hawkmoth pollination syndrome: they produce a strong, spicy-sweet scent, are pale in colour, have a long floral tube, and first open in the evening (Raguso and Pichersky, 1995). Nevertheless, they are additionally pollinated by a mix of bees, flies, diurnal moths, butterflies and hummingbirds. The other pollinators are able to access the nectar and/or pollen, and the plant takes advantage of the fitness contributions of these other pollinators in the face of substantial spatiotemporal variation in hawkmoth visitation (Miller *et al*., 2014). Here we show (B) a pollen-collecting bee unaffected by floral tube length and (C) a hawkmoth with a long proboscis that precisely fits the floral tube. (D) In *Tritoniopsis revoluta* (Iridaceae), nectar-foraging bees likely impose weak selection on floral tube length because nectar wells up the long tubes, making it accessible to the short-proboscid bees irrespective of floral tube length (Newman *et al.*, 2025). (E) Despite the importance of bee pollinators, *T. revoluta* tube length always matches the proboscis lengths of the long-tongued fly pollinators, even when they are uncommon (de Merxem *et al*., 2009; Anderson *et al*., 2014). Photos: K. Kay and B. Anderson. The image in panel D is from Anderson B, Ros P, Wiese TJ, Ellis AG. 2014. Intraspecific divergence and convergence of floral tube length in specialized pollination interactions. *Proceedings of the Royal Society of London: Series B, Biological Sciences* 281: 20141420, by permission of The Royal Society. It is not covered by the terms of the Creative Commons licence of this publication. For permission to reuse, please contact the Royal Society.

One of the longstanding conundrums in floral evolution is that flowers are often more generalized in their pollinators than their pollination syndromes would predict (e.g. Ollerton, 1996; de Merxem *et al*., 2009; Rosas-Guerrero *et al*., 2014; Pauw *et al*., 2020). An MEPP perspective might assume that the unpredicted pollinators are transient or ineffective, and not contributing to floral adaptation (e.g. Johnson and Steiner, 1997), or even functioning as nectar or pollen thieves (reviewed in Irwin *et al*., 2001; Hargreaves *et al*., 2009). In contrast, Aigner (2001) proposed another possibility, in which floral morphology can appear to match a less effective pollinator if it has a steeper fitness function (i.e. the less effective pollinator's contribution to fitness changes greatly with changes in floral phenotype), rather than the MEP per se. This scenario is likely if the less effective pollinator selects more strongly on floral traits than the MEP, such that it disproportionately contributes to the steepness of the combined fitness function (Fig. 2). Some pollinators can be extremely effective (contribute greatly to reproductive success) but select very little on particular floral traits (i.e. their contribution to fitness changes very little with changes in floral phenotype, resulting in a flat fitness surface). In contrast, other pollinators can contribute less to reproductive output but still select more strongly on floral traits (i.e. fitness changes quickly with changes in floral phenotype, resulting in a peaked fitness surface). Flat fitness surfaces allow plants to adapt to other (often less effective) pollinators without the trade-off of a loss in fitness. Importantly, the optimum floral phenotype, which combines the fitness surfaces of all pollinators, can closely match the optimum generated by the pollinator with the more defined peak, even if it is a far less effective pollinator overall (Fig. 2). In this case, an MEPP perspective would lead to confusion about pollinator-driven selection.

**Fig. 3.**
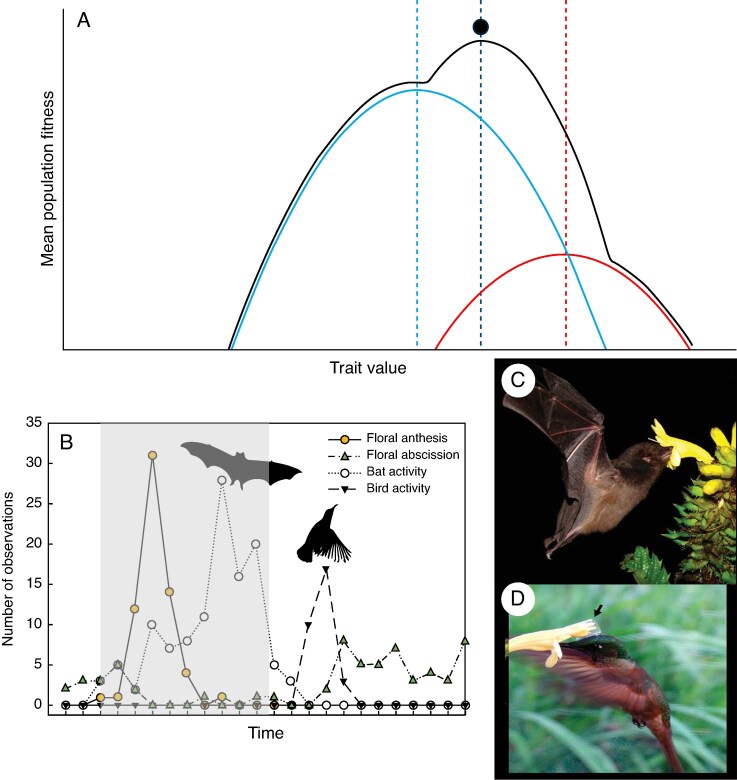
Intermediate phenotypic optimum facilitating a bimodal pollination system. (A) Plant populations can have an intermediate phenotypic optimum (black dot), which does not match the peak fitness contributions by either of the pollinators. Here, a flower with an intermediate floral phenotype gains fitness from the red and the blue pollinator and is fitter than a phenotype specialized to either pollinator. (B) A possible example of this are the flowers of *Kigelia africana*, which possess many traits associated with bat pollination (reproduced from Newman *et al*., 2021). Bats are the most effective pollinators, but the flowers are also pollinated by sunbirds. Instead of opening and closing at night as one may expect from a purely bat-pollinated flower, the flowers remain open for much of the daylight hours, allowing diurnal bird visitation. (C, D) Similar results are found in the South American species *Aphelandra acanthus*, which stays open both night and day, facilitating pollination by bats and hummingbirds (reproduced from Muchhala *et al*., 2009). The arrow in (D) points at a sticky tape used to estimate pollen receipt. Photos in panels C and D are reprinted from Muchhala N, Caiza A, Vizuete JC, Thomson JD. 2009. A generalized pollination system in the tropics: bats, birds and *Aphelandra acanthus*. *Annals of Botany* 103: 1481–1487, by permission of Oxford University Press. This content is not covered by the terms of the Creative Commons licence of this publication. For permission to reuse, please contact Oxford University Press.

Floral morphology may also not reflect the morphology of the MEP if the fitness peaks of different pollinators are close together and result in large combined contributions (Fig. 3). This scenario may result in phenotypic optima that are intermediate among those predicted by each pollinator contribution, even when one pollinator is more effective than the other (Dellinger *et al*., 2019; Newman *et al*., 2021). A typical study in such a population would find that one pollinator is more effective than the other, but that when a range of phenotypes is explored, the optimal floral phenotype would not ‘match’ either pollinator. In contrast, the MEPP assumes that adaptation is driven by the MEP, regardless of visitation by other pollinators (Stebbins, 1970), perhaps with the assumption that fitness peaks are steep and far apart and incur large fitness trade-offs when plants adapt to either pollinator (i.e. adaptation to one pollinator results in loss of fitness contributions from the other pollinator). In the case of a large valley between visitor-specific fitness contributions, selection should drive specialization to one or the other pollinator, depending on the initial phenotype and the relative peak heights. This is the scenario best explained by the MEPP, but fitness landscapes need not always have large valleys.

**Fig. 4.**
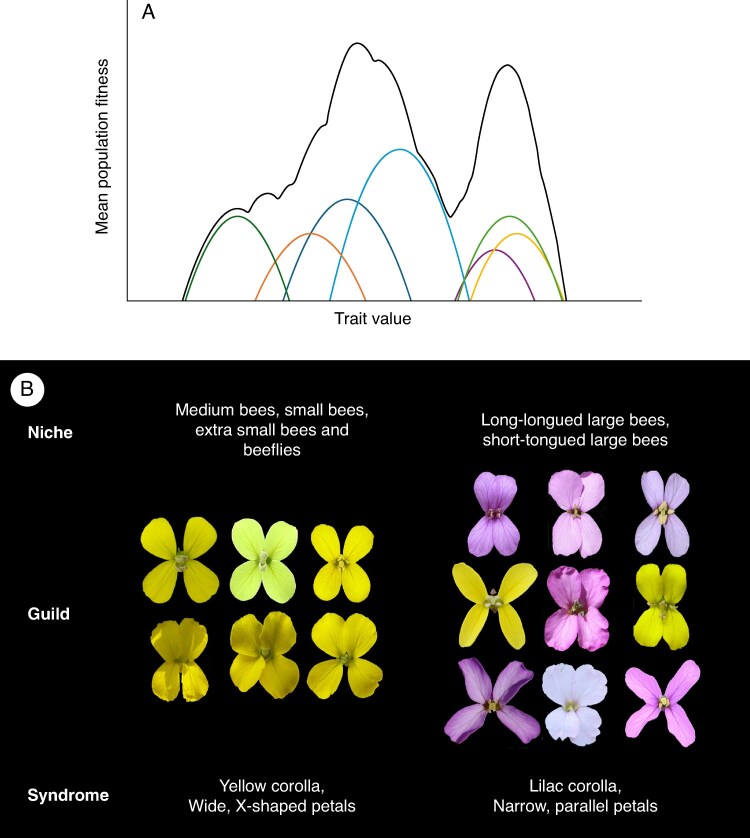
Adaptive landscapes for generalist flowers. (A) A hypothetical adaptive landscape produced by a community of pollinators for a generalist flower. The fitness contributions by each pollinator species are indicated by different colour lines, while the additive fitness function is shown in black. Here, the combined effects of different pollinators generate steep adaptive surfaces where peaks do not correspond to a single pollinator, but rather to two different generalist floral phenotypes that may be favoured in the community. (B) The genus *Erysimum* (Brassicaceae) shows a surprising array of floral variation for a genus assumed to have generalist flowers. Gómez *et al.* (2015) divided 40 species into five different generalist pollinator niches and demonstrated that these niches were associated with different floral phenotypes or syndromes. Here, we show the species belonging to two of these generalist pollinator niches. Species pollinated by medium, small, extra small bees and beeflies tend to have yellow corollas with wide X-shaped petals, whereas species pollinated by various species of large bee tend to have lilac corollas with narrow, parallel petals. Panel B adapted from Gómez JM, Perfectti F, Lorite J. 2015. The role of pollinators in floral diversification in a clade of generalist flowers. *Evolution* 69: 863–878, by permission of Oxford University Press. This content is not covered by the terms of the Creative Commons licence of this publication. For permission to reuse, please contact Oxford University Press.

There may be a perception that selection is much stronger in highly specialized systems, because in generalized systems each visitor-specific contribution appears minor, indistinct and potentially conflicting. If contributions by different pollinators cancel one another out, selection on floral traits may be weak (Sahli and Conner, 2011). However, multiple empirical examples now suggest that generalist plants are locally adapted to particular communities of pollinators. Using an adaptive landscape framework, it becomes apparent that different pollinators in generalized pollination systems may also produce very steep surfaces if they are additive, leading to adaptive generalization (Fig. 4). In such systems where many pollinators may have overlapping fitness contributions, optimum floral phenotypes do not necessarily correspond to any particular pollinator, and high degrees of specialization are not a prerequisite for adaptation in floral phenotype (Sahli and Conner, 2011). In such situations, which adaptive peak is climbed may depend on the starting phenotype. Overall, the floral phenotype with the highest fitness in a population is going to depend on the local assemblage and availability of pollinators, and the phenotypic range and slope of their fitness contributions. Flowers that appear specialized at first glance may not be, and many may show adaptive generalization (Ohashi *et al*., 2021).

### The presence of one pollinator can alter the fitness contributions of another

In the above scenarios, visitor-specific fitness contributions neatly sum to produce an overall fitness function. However, empirical and modelling studies suggest that this assumption of additivity is often violated (Thomson and Thomson, 1992; Aigner, 2001; Thomson, 2003; Ohashi *et al*., 2021). Thomson and Thomson (1992) and Aigner (2001) identified two reasons for non-additivity of visitor-specific fitness contributions. First, because rewards, pollen and ovules are all finite, there are upper bounds to both female and male fitness. Consequently, adding visitor-specific fitness contributions may exceed the total possible fitness. For example, boundaries imposed by ovule number may make pollen deposition a poor measure of female fitness because a stigma can receive many more pollen grains than it has ovules to fertilize. Second, pollinators may vary in their pollen transfer efficiency and/or offspring quality, such that pollination by one pollinator can preclude pollination by another visitor with a higher or lower fitness contribution. Moreover, these issues may operate in the same system, such that pollinators differing in quality can interact within a bounded fitness landscape.

Thomson (2003), in discussing bee and hummingbird pollination of *Penstemon*, used the analogy of pollinators as leaky buckets moving water (pollen) from one pond (flower) to another. The leaky buckets (pollinators) deposit only a fraction of the pollen that they collect, and they may differ in terms of their leakiness (wastefulness). For example, bees may be viewed as very leaky buckets because of their propensity to groom and consume pollen, whereas hummingbirds may be viewed as less wasteful pollinators. Having very leaky bucket pollinators in addition to higher quality pollinators is only beneficial when there is a lot of water to move (pollen is plentiful). However, as pollinators become more plentiful, pollen becomes more limited, and wasteful pollen removal will impose a cost, or opportunity trade-off (*sensu* Ohashi *et al*., 2021) on plants if that pollen could have been removed by a less wasteful pollinator. Thus, the same pollinator can vary from mutualist to parasite depending on their numbers and the availability of other pollinators in the community. In *Penstemon*, if both bees and hummingbirds visit and bees function as a leakier bucket because of grooming, they may become parasites and drive selection for ‘anti-bee’ filtering traits (Castellanos *et al*., 2004).

Thomson (2003) conditioned his leaky bucket analogy on there being a finite amount of pollen; however, it can operate whenever fecundity is bounded, including through female function and offspring quality (Ohashi *et al*., 2021). For example, Thompson and Cunningham (2002) found that when *Lithophragma parviflorum* (Saxifragaceae) populations are only pollinated by brood-parasitic *Greya politella* moths, the relationship is mutualistic, but when populations are also visited by bombyliid flies that do not parasitize ovules, the relationship between *Greya* and *Lithophragma* becomes parasitic. This type of phenomenon could also happen when pollinators differ in their movement patterns and consequently the quality of offspring produced by their visits. In that case, the presence of a pollinator contributing higher quality offspring could render the other pollinator's fitness contributions negative.

**Fig. 5.**
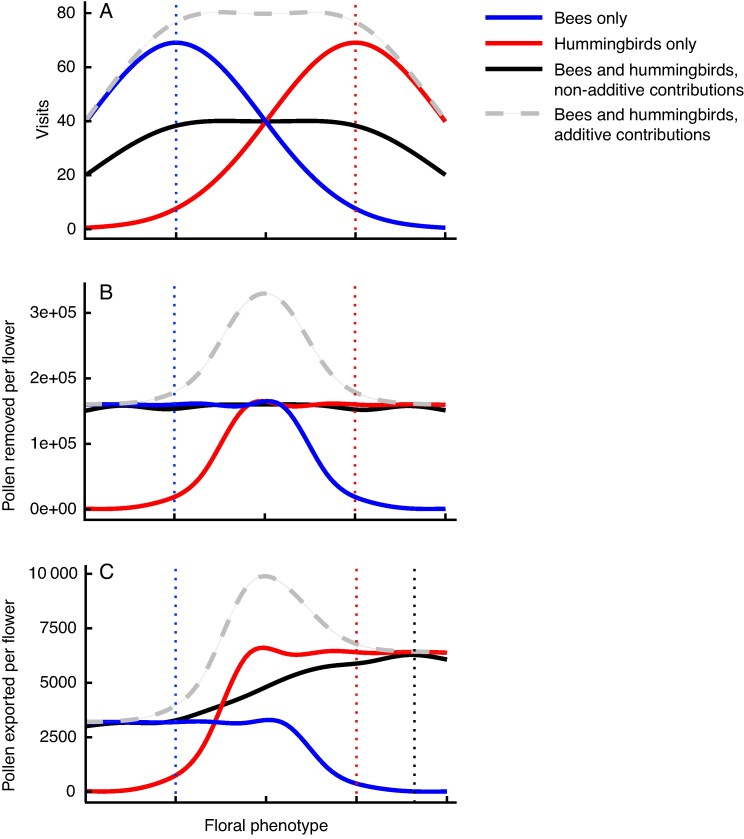
The presence of one pollinator can decrease the fitness contribution of another, such that total fitness is not a simple sum of visitor-specific fitness curves. Here, we model floral adaptive landscapes for three measures of fitness (visitation, pollen removed and pollen export). We simulate visitation by bees and hummingbirds to a population of 100 flowers with a uniform distribution of phenotypes spanning both a bee and hummingbird optimum for attraction (visitation probability) and fit (pollen removal per visit), indicated in panels above with blue and red vertical dotted lines, respectively. Both pollinators have a 90 % visitation probability to their optimum phenotype and 10 % to the other phenotype, with the probabilities approximating a normal distribution. Similarly, both pollinators remove 10 000 pollen grains per visit from their optimum phenotype and 2500 from the other phenotype, again with pollen removal normally distributed. Bees subsequently deposit 2 % of the pollen they remove per visit, whereas hummingbirds deposit 4 %, with the rest of the pollen lost from the system. Each flower produces 160 000 total pollen grains. We ran the model ten times, with 3500 total visits in each iteration for bees only, hummingbirds only and mixed environments in which bees and hummingbirds were equally abundant, and we sampled for visits with a binomial process. For comparison with our interactive model, we also present additive results at each stage. Simulations were run in R 4.0.5 (R Core Team, 2021) and smoothed results plotted with ggplot2 (Wickham, 2016). (A) In a mixed pollinator environment, visits show opportunity trade-offs that flatten the distribution of visits across phenotypes compared with additive contributions and show lower visitation to optimal phenotypes for bees and hummingbirds compared with when either pollinator is alone. Trade-offs in visitation could result from direct competition for flowers or from visitation reducing future visitation, perhaps through depletion of rewards or trait changes. (B) In a mixed pollinator environment, essentially all pollen is removed across all floral phenotypes, flattening the distribution. Adding pollen removal per flower from single-pollinator environments produces an intermediate optimum that exceeds pollen production per flower and is unrealistic when pollen removed by one pollinator cannot be removed by another. (C) Pollen exported per flower is affected by the symmetrical opportunity trade-offs at preceding stages (visitation, removal) and the asymmetrical opportunity trade-offs for export because of higher pollen transfer efficiency for hummingbirds compared with bees. In isolation, bees have positive fitness contributions across a range of phenotypes. However, in a mixed visitor environment, pollen export per flower is highest for a phenotype that is more extreme than the hummingbird optimum (black dotted line) because it filters out the lower-quality bee pollinators. Moreover, simply adding the bee and hummingbird curves misleadingly shows optimal pollen export per flower at an intermediate phenotype. Model development by Arjan Engelen.

Considering non-additive visitor-specific fitness contributions is critical to understanding both adaptive generalization and pollinator shifts. Aigner (2001) modelled interactions between visitor-specific fitness contributions with a constant interaction strength that was not scaled to variation in the visitation rate of different pollinators across phenotypes, producing some results that are hard to interpret. More recently, Ohashi *et al*. (2021) modelled these interactions in a more flexible way, with the interactive effects scaling to visitation rates, but only for fixed floral phenotypes. Here, we extend these efforts by modelling floral adaptive landscapes while incorporating variation in visitation rate and pollinator fit across a range of floral phenotypes in a two-visitor system in which the pollinators differ in pollen export efficiency (Fig. 5). We show that bounding both the number of visits and pollen grains flattens the total visitation and pollen removal fitness curves, respectively, compared with additive effects (Fig. 5A, B). Moreover, differences in pollen export efficiency interact to produce an extreme phenotypic optimum that excludes the less efficient pollinator, whereas the additive phenotypic optimum is intermediate between visitor-specific optima (Fig. 5C). We suggest that both modelling and empirical efforts to understand non-additive pollinator fitness contributions are important areas where progress can be made in understanding the evolution of floral traits.

### Fitness as the currency of selection

Accurate measurements of fitness are important for us to gauge the strength, direction and shape of fitness landscapes. However, because of the difficulties in measuring fitness properly, biologists usually measure components of fitness (Box 1). Different components of fitness can lead to very different conclusions about floral adaptation, potentially leading to incorrect conclusions about pollinator-driven selection on floral traits (Fig. 5). On the other hand, breaking down natural selection into its various components provides useful information about both the agents of selection and targets of selection. Below, we outline some aspects of pollinator-driven selection for which we have a relatively poor understanding, including male fitness and offspring quality.

### Floral fitness has a male and female component

**Fig. 6.**
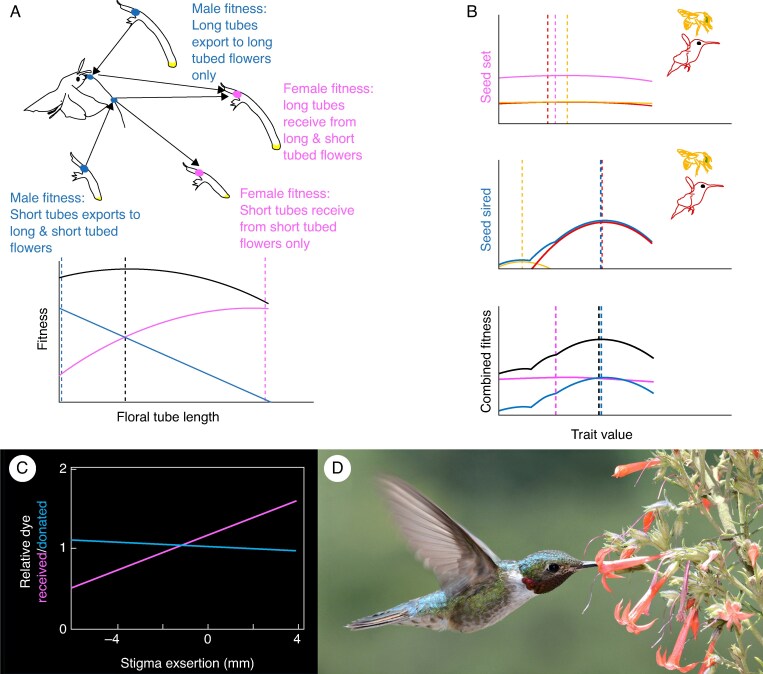
Male and female fitness components may show selection on different floral traits and/or for different trait optima. (A) In this hypothetical example, short-tubed flowers are better at exporting pollen, but long-tubed flowers are better at receiving pollen (Ellis and Johnson, 2010). Male (blue) and female (pink) fitness components select for different floral tube lengths, but the optimal floral tube length reflects a trade-off between the two (black dashed line). Here, quantification of only male or female fitness components would generate misleading fitness landscapes. (B) Different pollinators (e.g. birds and bees) may have similar effects on the female fitness component (pink) if there is little pollen limitation in this hypothetical example (top panel). However, birds may be more efficient at exporting pollen than bees (middle panel). The optimal floral phenotype (black dashed line) is a product of both male and female fitness functions (bottom panel), but because the female fitness function is relatively flat, the optimum is determined primarily by the male fitness component. Here, quantifying the female fitness component alone would generate a misleading fitness landscape. (C) A possible example of (A) was found for stigma exsertion in *Ipomopsis aggregata* (redrawn from Campbell, 1989), where more exserted stigmas were associated with greater numbers of pollen grains received (female fitness) but fewer pollen grains donated (male fitness). (D) *Ipomopsis aggregata* being visited by a broad-tailed hummingbird. Photo: David Inouye. Panel C is redrawn from Campbell DR. 1989. Measurements of selection in a hermaphroditic plant: variation in male and female pollination success. *Evolution* 43: 318–334, by permission of the Society for the Study of Evolution. This content is not covered by the terms of the Creative Commons licence of this publication. For permission to reuse, please contact the Society for the Study of Evolution.

Accurately describing floral adaptive landscapes for hermaphroditic plants requires expanding measures of fitness to include siring success, or male fitness (Box 1). Although most studies of pollinator-driven selection only measure components of female fitness, such as pollen deposition and seed set, male and female fitness may sometimes select for different floral phenotypes (e.g. Ellis and Johnson, 2010; Fig. 6). Moreover, selection may frequently be stronger through male function than through female function (Bateman, 1948; Ashman and Morgan, 2004). Because ovules are finite in number and pollen grains numerous, female fitness is often thought to be more limited by resources than by mating opportunities (pollinator visits), relative to male fitness (Janzen, 1977; Willson, 1994). Consequently, full seed set may be achieved with limited investment in attraction or reward traits and by a wide variety of floral phenotypes, resulting in a relatively flat fitness function (Fig. 6B). In contrast, male fitness, or siring success, is often limited by access to and competition for mates (Bateman, 1948; but see Wilson *et al*., 1994) and can show large variance and steep floral adaptive landscapes. Consequently, traits involved in pollinator attraction (e.g. flower size, colour, scent and reward) and mechanical fit (e.g. shape and size) are sometimes thought to have evolved primarily through the male fitness pathway (Willson, 1979). The few studies that have compared male and female selection pathways often find evidence for Bateman's principle (that male fitness is more variable and driven by access to mates), but so far there is little evidence to suggest that selection through male fitness is generally stronger (e.g. Hou *et al*., 2024). This lack of evidence may partly be due to the fact that male success is a complex and stochastic process (Inouye *et al*., 1994; Minnaar *et al*., 2019*a*) and that a large component of success may come down to luck, which is not heritable (Wilson *et al*., 1994). In reality, the relative strength of selection through either male or female function is likely to be strongly affected by pollen limitation. The relative importance of the female fitness pathway is expected to be at its greatest when pollen is limited (63 % of studies; Knight *et al*., 2005), because then female fitness will be most variable. However, as greater proportions of ovules are fertilized, the relative influence of male fitness is likely to increase. In fact, competition among pollen grains for a limited number of ovules is likely to be at its strongest when pollen is plentiful and most ovules are fertilized (when selection through the female fitness pathway is at its weakest). Furthermore, different pollinators may vary in their contributions to pollen export, even when their contributions to seed set are similar (Fig. 6B). An inclusive view of male and female fitness suggests that some floral traits may represent trade-offs between what is best for male and female fitness and that these trade-offs may explain apparent mismatches between pollinator morphology and fitness when only one component is measured. Other traits, such as gradual pollen presentation and explosive pollination, can only be understood by studying aspects of male fitness (e.g. Harder and Thomson, 1989; Kay *et al*., 2020; Anderson *et al*., 2024). Newer tools, such as quantum dots and molecular markers that do not require species-specific development, should increase the tractability of studying male fitness and help identify situations where male and female floral adaptive landscapes differ (Austen and Weis, 2016; Minnaar and Anderson, 2019).

### Not all offspring are created equal

Although rarely assessed, fitness contributions of different pollinators depend not just on the quantity of seeds set and sired, but also on their quality (Box 1). Offspring quality can be affected by inbreeding (Lloyd, 1992; Lloyd and Schoen, 1992) and outbreeding depression (Fenster and Dudash, 1994; Dudash and Fenster, 2000) and by pollen competition and multiple paternity (Marshall and Ellstrand, 1986). Because of variation in pollinator foraging patterns, mobility and pollen carryover, the offspring contributed by different pollinators could vary greatly in quality. Optimal outcrossing distance will be specific to the plant population and depend on such things as inbreeding depression, spatial genetic structure and the scale of local adaptation (Waser and Price, 1989; Krauss *et al*., 2017). For example, a more territorial pollinator could cause high offspring production but with high geitonogamy and biparental inbreeding, whereas another highly mobile pollinator could cause fewer, but highly outcrossed progeny. The fitness contribution of the territorial pollinator may be greater in plants with low inbreeding depression, strong local adaptation and/or genetic incompatibilities among populations, whereas the fitness contribution of the mobile pollinator would be greater in plants with high inbreeding depression and less population structure. In a striking example, Betts *et al.* (2015) showed that the Neotropical understorey herb *Heliconia tortuosa* appears to recognize visits by trap-lining hermit hummingbirds associated with higher quality offspring and selectively facilitate pollen tube growth, suggesting the importance of offspring quality in floral adaptation. Pollinator behaviour can also affect pollen carryover, with subsequent impacts on pollen dispersal distances and the composition of pollen loads. Mixed pollen loads from multiple sires can increase pollen competition, opportunities for female choice, and genetic diversity among offspring (Karron *et al*., 2012; Valverde *et al*., 2019). Small insects tend to be less mobile than larger insects or vertebrates, with resulting differences in gene flow and population genetic structure (Gamba and Muchhala, 2020, 2023). Grooming pollinators, such as bees, typically have low pollen carryover (Castellanos *et al*., 2003; Holmquist *et al*., 2012) compared with those that do not systematically groom and consume pollen. Nevertheless, because of the difficulty of continuing pollination studies into the next generation, few have directly assessed the quality of offspring produced by visits of different pollinators (but see Herrera, 2000; Gómez, 2000). For example, Herrera (2000) demonstrated that *Lavandula* seedling mortality was higher for progeny resulting from large bee pollination than for progeny resulting from small bee and butterfly pollination.

Demographic studies show that plants vary somewhat predictably in the relative importance of different fitness components to total fitness, and we can use these insights to predict when it will be most important to incorporate inferences of offspring quality into studies of pollinator-driven selection. For annual plants, especially colonizing or weedy species, fecundity (or offspring number) is likely to be the best fitness surrogate (Ramula *et al*., 2008). The extreme of this spectrum is occupied by highly selfing annual weeds that produce large numbers of offspring without the aid of pollinators. In contrast, for perennial, iteroparous, and woody plants, especially those in stable environments, total fitness is expected to be more sensitive to survival than fecundity (Silvertown *et al*., 1993; Crone, 2001). In these cases, the quality of offspring produced may be more important than the quantity. These plants are also likely to have few, well-provisioned seeds, and not be limited by pollen quantity (Aizen and Harder, 2007). Meta-analysis of inbreeding depression mirrors these patterns, in that longer-lived and woody plants tend to have higher inbreeding depression than annuals and herbaceous plants (Angeloni *et al*., 2011). Consequently, offspring fitness of long-lived/woody plants should be most affected by differences in pollinator foraging patterns and their propensity to result in selfing or biparental inbreeding. Incorporating estimates or predictions of offspring quality into floral adaptive landscapes is rarely done, but may be important for understanding the relative fitness contributions of different pollinators.

### Temporal variation in the local pollinator climate will affect floral adaptation

Pollinator abundances and the co-flowering plant community may both vary over time for a plant population (CaraDonna *et al*., 2021), leading to variation in the phenotypic optimum among years or even within a season. In general, temporal variation should select for the phenotype with the highest geometric mean fitness over time (Haldane and Jayakar, 1963; Gillespie, 1973), which is particularly sensitive to low values. Temporal variation may be most important to consider when particular functional groups of pollinators have large changes in visitation over time. In that case, even if they have high fitness contributions in good years, selection is unlikely to favour exclusive pollination by this group, at least for short-lived plants. Rather, we might expect compromise phenotypes that allow fitness contributions from temporally unreliable but highly effective pollinators, alongside temporally stable fitness contributions from more reliable pollinators. Alternatively, flowers may use multiple traits (including trade-off mitigating traits) to maximize fitness across the temporal variability of functionally different pollinators (e.g. Ohashi *et al*., 2021). Consequently, single-season studies to identify the MEP may come to different conclusions based on the year of the study, ranging from omitting an important pollinator entirely to concluding that the population is not adapted to the MEP. While it is becoming more common in plant–pollinator interaction studies to incorporate variation across the flowering season, multi-year studies are relatively rare, even though they may be most pertinent to understanding evolutionary dynamics. Moreover, these studies generally focus on community network structure, not the selective landscape for individual plant species interacting with functional groups of pollinators. Multi-year and across-season network studies may help us predict the types of pollinators and environmental conditions that are likely associated with temporal variation in pollinator-driven selection.

### Floral adaptive landscapes present both challenges and opportunities

In this section, we hope to demonstrate that pollinator-driven selection on floral traits within a plant population is both more complex and more interesting than implied by the MEPP. By using adaptive landscapes that incorporate all pollinators and nuanced considerations of fitness components, we gain a better understanding of how selection influences floral phenotype. Floral adaptive landscapes may be unexpected, complex in shape, and best visualized using splines, rather than simple linear or quadratic regression (Lande and Arnold, 1983; Morrissey and Sakrejda, 2013).

Embracing floral adaptive landscapes will require better empirical estimates of pollinator-driven selection. Specifically, we need to isolate the relative fitness contributions of different pollinators, through both female and male fitness pathways, across a broad range of floral phenotypes. Although estimating total lifetime fitness is often the aim for selection studies, it may be important to decompose total fitness into various components to understand the pollinator component, the traits targeted by pollinator-driven selection, and how pollinator-driven selection aligns or contrasts with total fitness. Some studies have managed to broadly disentangle the pollinator component by subtracting selection gradients of plants that received supplementary pollen from those estimated by open-pollinated controls (Sletvold *et al*., 2010); however, less is known about the contributions made by different pollinators. To this end, studies have separated different contributions into night and daytime contributions (Sletvold *et al*., 2012; Miller *et al*., 2014; Torres-Vanegas *et al*., 2024), experimentally excluded or included certain pollinators (e.g. Bischoff *et al*., 2015; Bezemer *et al*., 2019; Wawrzyczek *et al*., 2025), or quantified pollen grains deposited during single visits by different pollinators (e.g. Page *et al*., 2021). One frequent problem with selection studies is that if selection is strong, there will often be little trait variation within a population, allowing us to assess only a narrow window of the adaptive landscape. In order to understand these landscapes more broadly, trait variation can be enhanced by translocating flowers from other populations (e.g. Galen, 1996; Newman *et al*., 2025), directly manipulating phenotypes (e.g. Fulton and Hodges, 1999), generating recombinant hybrids (e.g. Schemske and Bradshaw, 1999), using natural hybrid zones (e.g. Campbell, 2004), or using model flowers (e.g. Schemske and Ågren, 1995). Less attention has been paid to accurate estimates of the male fitness contributions of different pollinators or to the quality of offspring produced. These aspects of fitness are critical for accurate depictions of adaptive landscapes and for assessing how the fitness contributions of individual pollinators may interact in non-additive ways. With enough studies of floral adaptation within populations, we may begin to understand certain rules about different types of pollinators. For example, there may be systematic patterns when comparing different pollinator types, such as grooming versus non-grooming, obligate versus facultative flower feeders, highly mobile versus less mobile, and territorial versus non-territorial. Finally, we need to turn our attention to how these fitness landscapes differ geographically in order to understand how pollinators drive divergence of floral form.

## HOW DOES FLORAL DIVERGENCE HAPPEN?

**Fig. 7.**
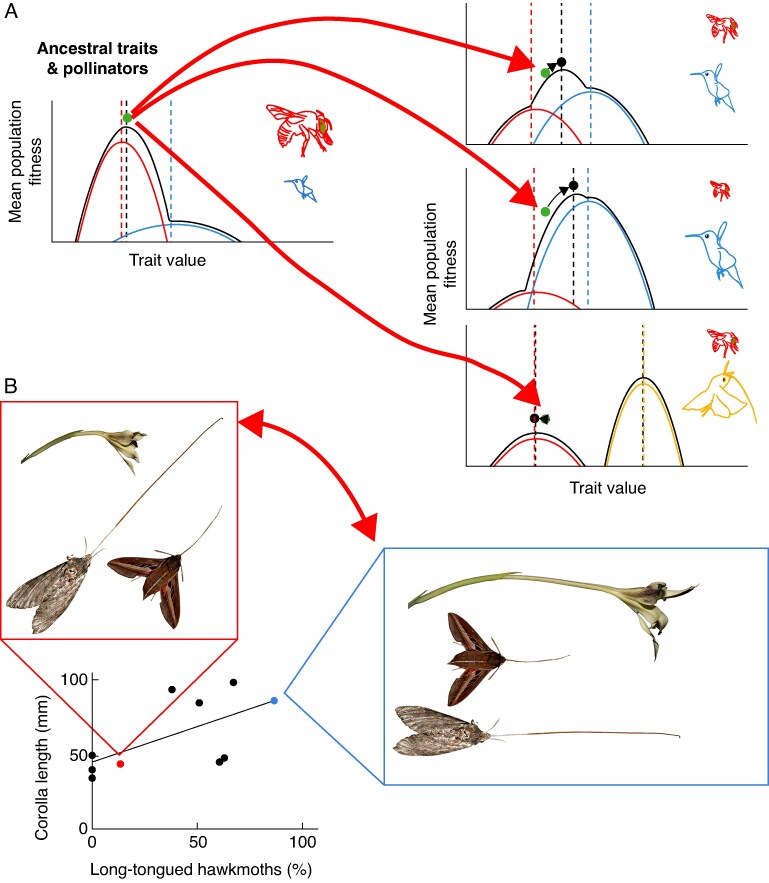
Stebbins (1970) envisaged how the traits of an ancestral population may shift and adapt to a novel pollinator. Here we depict a slightly more complex scenario where floral morphology adapts to changes in a pollinator community of two pollinators. Such changes can occur as a result of *in situ* temporal variation in the pollinator landscape or of spatial variation in the pollinator landscape when individuals from an ancestral population colonize a novel community. (A) A depiction of three different changes in the pollinator landscape that vary in severity. Relative size of the pollinators represents their differences in potential effectiveness. A subtle change in the relative importance of bird and bee pollinators may result in changes to floral morphology, but those changes may not match the traits predicted by either pollinator alone (also see Fig. 3). Large-scale changes in the relative importance of bird and bee pollinators may result in what we interpret as an almost complete shift from one syndrome to another. Importantly, in both of these scenarios, the least effective pollinator (bee) in the novel population still selects on the floral trait (also see Fig. 1), assuming fitness contributions are additive (see Fig. 5 for an alternative scenario). Alternatively, plants may not be able to adapt to a complete shift in the pollinator landscape if the fitness contribution of the novel pollinator (here, a moth) does not overlap with the ancestral phenotype. (B) A putative example of subtle shifts in the pollination climate driving floral divergence may be *Gladiolus longicollis*, which is visited by both short- and long-tongued hawkmoths in most populations (Anderson *et al*., 2010). Floral tubes appear to vary geographically and population averages appear to be associated with which moths are most abundant. In this example, it is not clear which traits are ancestral or derived. Photos: B. Anderson. The graph in panel B is redrawn from Anderson B, Alexandersson R, Johnson SD. 2010. Evolution and coexistence of pollination ecotypes in an African *Gladiolus* (Iridaceae). *Evolution* 64: 960–972, by permission of the Society for the Study of Evolution. This content is not covered by the terms of the Creative Commons licence of this publication. For permission to reuse, please contact the Society for the Study of Evolution.

Given that essentially all plants specialize on a subset of available pollinators and that flowering plants have been adapting to pollinators for over 100 my, we need to be able to explain how a population or species exhibits one adaptive phenotype and then shifts to another. The most striking case is when a plant lineage shifts from one pollination syndrome to another, which we know has occurred many times in the history of angiosperms (reviewed in Rosas-Guerrero *et al*., 2014). The process of pollination shifts has been perplexing because it often appears that closely related species have floral traits with clear phenotypic trade-offs in attraction and fit with different pollinators. Without invoking a large role for genetic drift, how does a population traverse such a valley? Stebbins hypothesized that pollinator shifts could be driven by large decreases in pollinator abundance as a plant species colonizes new habitats, stating ‘The evolutionary shift from one vector to another is probably trig­gered by the entrance of the plant into a habitat where the original vector is scarce and the new vector is abundant’ (Stebbins, 1970) (Fig. 7). While Stebbins's statement makes it sound as though substantial and nearly qualitative changes in the pollinator landscape cause floral divergence, we wonder whether divergence may be possible after more subtle changes in the pollinator landscape. Such changes may allow adaptation to new pollinators even when the ancestral pollinators are abundant within the landscape. Others have invoked mutations of large effect that would allow a plant population to rapidly traverse a fitness valley. However, single large-effect loci are insufficient for shifts between multivariate syndromes and still need to spread through a population by selection. Although these explanations may sometimes apply, Thomson (2003) and Thomson and Wilson (2008) carefully discuss problems with both of them. Here, we argue that pollinator shifts become easier to understand by using an adaptive landscape perspective that accurately assesses the breadth, height, and interactions among visitor-specific fitness contributions.

### Visitors may become ‘scarce’ in some regions without being absent

**Fig. 8.**
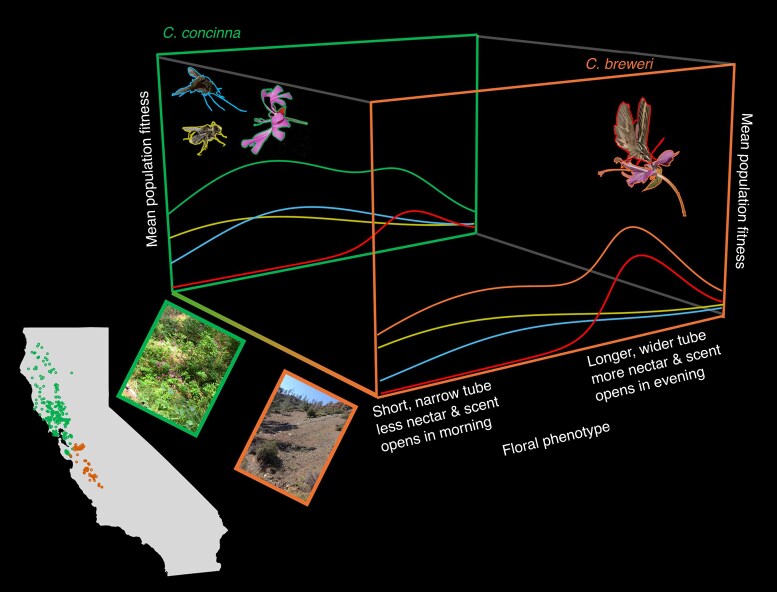
Hypothetical floral adaptive landscapes for sister species of *Clarkia* across an ecogeographic range in California. *Clarkia concinna* and *C. breweri* are sister species with adjacent ranges across a sharp North–South/maritime–inland climate and vegetation gradient in the coast ranges of California (Goff *et al*., 2021). *Clarkia concinna* is pollinated by bees, flies, diurnal Lepidoptera and occasional hummingbirds (Miller and Kay, 2025), whereas *C. breweri* exhibits a hawkmoth pollination syndrome and is pollinated by nocturnal hawkmoths along with a similar mix of diurnal pollinators. Here, we show hypothetical fitness functions for a subset of pollinator functional groups (bees in yellow, flies in blue and hawkmoths in red). Reciprocal translocations of flowering *Clarkia* plants showed that the diurnal insects, although present, are less common in the dry, open habitat of *C. breweri* compared with the mesic, forested habitat of *C. concinna*, helping to explain the shift to the hawkmoth pollination syndrome (Miller *et al*., 2014). Moreover, hawkmoths did not visit *C. breweri* when it was placed in *C. concinna* habitat, even though hawkmoths are known to pollinate other plants in the area. Photos: K. Kay.

Floral divergence is most frequently depicted as the result of geographic variation in pollinator communities, which can generate geographic mosaics in the shapes of local fitness landscapes (Herrera *et al*., 2006). Evidence for these clean-cut geographic mosaics in selection can be found for highly specialized pollination systems where the disappearance of one important pollinator in part of a plant's range drives an adaptive shift to a novel pollinator (Newman *et al*., 2015; Trunschke *et al*., 2019). However, more subtle differences in the pollinator assemblages of more generalist plants can also cause floral divergence between different populations. For example, sister species *Clarkia concinna* and *C. breweri* show marked floral divergence despite a largely overlapping set of pollinator types that vary in visitation rate across their combined geographic range (Fig. 8; Miller *et al*., 2014). This work shows that differences in habitat affinity and the impacts these have on pollinator assemblages (and possibly the co-flowering community; see below) can drive floral divergence even without specialization on single functional groups or the disappearance of an important pollinator from some regions.

**Fig. 9.**
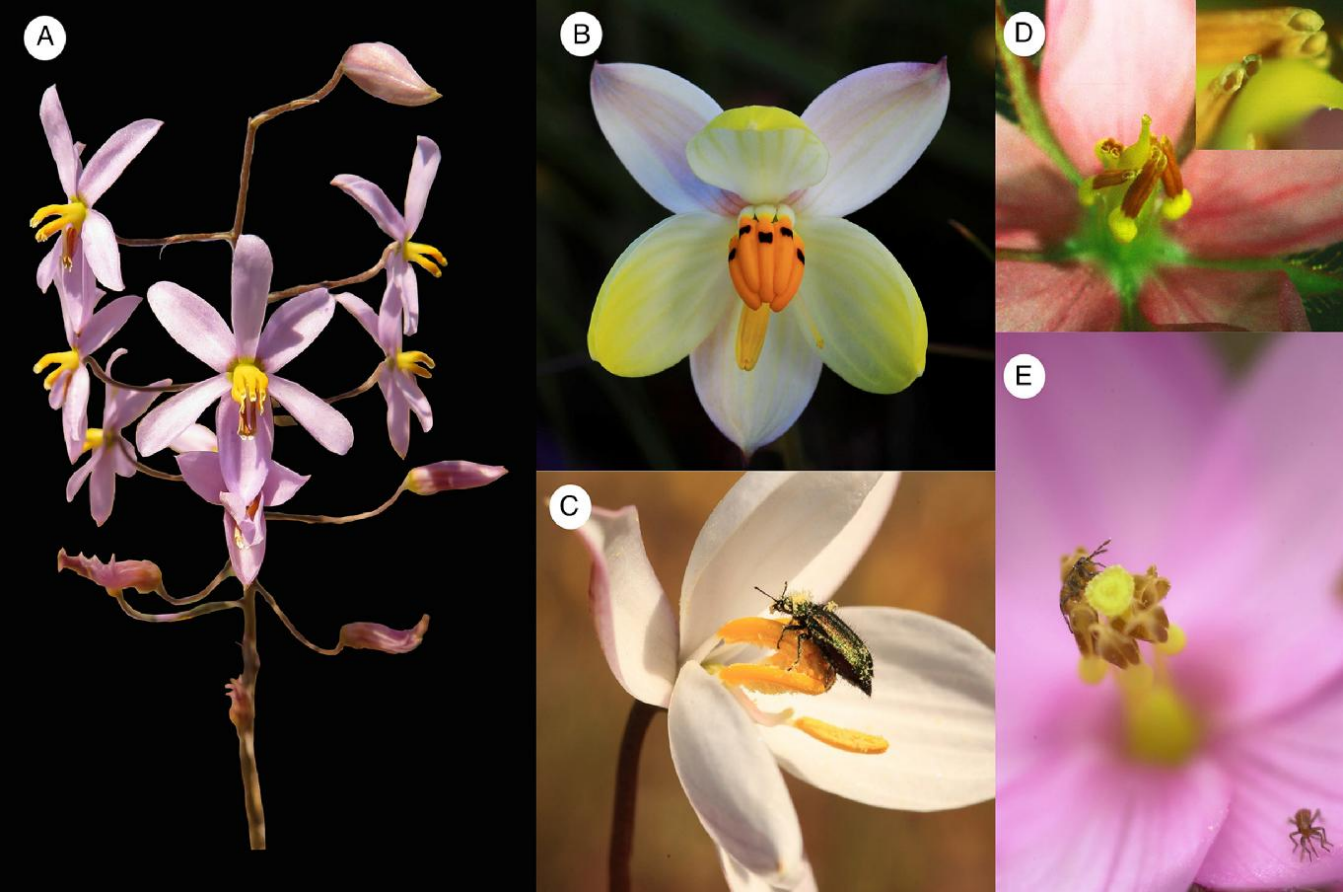
Competition for pollinators may drive the evolution of novel pollination strategies in South African buzz-pollinated plants. (A) Bee visits to *Cyanella hyacinthoides* are reduced in communities with other rewarding species. (B) Similarly, *Cyanella alba flavescens* plants are critically pollen-limited because buzz pollinators seldom visit, despite their presence in all populations. (C) This appears to have had evolutionary consequences in some populations of *Cyanella alba alba* that have evolved laterally dehiscent anthers, allowing more effective pollen transfer by non-buzzing pollen feeders. (D) The poricidal anthers and lack of nectar in *Roridula dentata* also suggest buzz pollination (see insert). Although buzz-pollinating bees frequently occur in *Roridula* populations, buzz pollination has seldom been observed, and the exclusion of bees does not affect seed production. Pollination is performed primarily by mutualistic hemipterans that live exclusively on these plants. (E) Poricidal anthers in the sister species, *Roridula gorgonias*, appear to have been secondarily lost, attracting greater numbers of hemipterans. Photos: J. Kemp, B. Anderson, C. Ewart-Smith.

Mosaics in the fitness landscape may also occur as a result of variation in the co-flowering plant communities rather than, or in addition to, changes to the pollinator community per se. For example, the co-flowering community can affect visitation rates (Albor *et al*., 2019) by different pollinators through processes of facilitation (e.g. Duffy and Stout, 2011) and interspecific (e.g. Feinsinger *et al*., 1991; Hanoteaux *et al*., 2012) or intraspecific (e.g. Ward *et al*., 2013) competition. Consequently, even if pollinator communities remain constant, their influence on the evolution of focal plants may change if floral communities change over space or time. Fluctuations in co-flowering communities may be short-term (e.g. successional or cyclical responses to weather), which may not be consistent enough to have lasting effects on floral evolution. However, plant community responses to long-term variations (e.g. climate differences) may cause more permanent shifts in fitness landscapes that result in transitions between pollinators and their associated syndrome traits. For example, in South Africa, competition for pollination services appears to have left several putatively buzz-pollinated species without pollinators or with very low visitation rates in parts of their ranges even though legitimate pollinators are present (Fig. 9). Buzz-pollinated plants usually only produce a pollen reward, which is hard for most pollinators to access, potentially making other plant community members much more attractive to visit. Kemp *et al.* (2022) demonstrated that bee visits to *Cyanella hyacinthoides* decreased as the relative abundance of other flowers increased, suggesting that competition with co-occurring plant species affects visitation. Such competition may also have evolutionary consequences, as evidenced by the apparent loss of the poricidal anthers associated with buzz pollination in *Cyanella alba* (Barrett and Fairnie, 2024; Paudel *et al*., 2024) and *Roridula* (Anderson *et al*., 2003).

### Overlapping visitor-specific fitness contributions facilitate pollinator shifts

As we describe above, even plants that appear to have specialized traits are often somewhat ecologically generalized. Apparent ‘secondary pollinators’, or those not matching the plant's pollination syndrome, are common and can even be highly effective (Rosas-Guerrero *et al*., 2014). Secondary pollinators present opportunities for shifts between pollination syndromes when they have non-zero fitness contributions, and the opportunity is pronounced if those pollinators have the potential for higher fitness contributions due to pollination quality (e.g. pollen transfer efficiency or offspring quality), which may often be the case in transitions away from pollen consumers, such as bees. In such cases, even subtle increases in abundance can raise their visitor-specific fitness contributions high enough to drive adaptation to a higher peak (Figs 7A and 5C). This idea echoes Stebbins’s (1970) hypothesis that pollination shifts happen through ‘selection along lines of least resistance’, using traits and pollinators that are already present, and the ‘transfer of function via an intermediate stage of double function’, in which both the ancestral and derived pollinators are capable of functioning. However, his language about the MEPP dismisses the importance of secondary vectors in driving selection on floral traits, leaving a disconnect between how he viewed selection within populations and divergence between lineages.

**Fig. 10.**
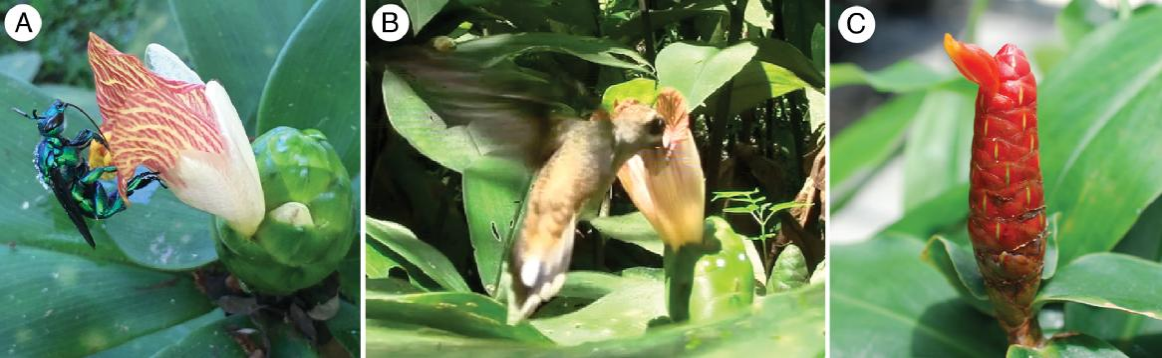
Hummingbirds can have broader fitness contributions across phenotypes than might be assumed from pollination syndromes. For example, *Costus malortieanus* is primarily pollinated by orchid bees and exhibits traits consistent with a typical orchid bee pollination syndrome for the genus, but is commonly visited by hermit hummingbirds late in the flowering season when other hummingbird floral resources are scarce. (A) An *Exaerete* sp. orchid bee uses the expanded labellum as a landing pad and the red and yellow nectar guides to orient itself before crawling inside the large gullet flower to nectar and receiving pollen on its thorax. (B) A *Phaethornis longirostris* hummingbird visits *C. malortieanus* and contacts the anthers and stigma with its bill despite the typical bee syndrome floral traits. (C) For comparison, *Costus scaber* is primarily pollinated by *P. longirostris* and shows characters typical of a hermit hummingbird syndrome in the genus, including loss of the landing pad and nectar guides, a short narrow flower, less concentrated nectar and brightly coloured floral bracts. Photos: R. Maguiña and K. Kay.

The Neotropical spiral gingers (genus *Costus*) provide an example of how less frequent or effective pollinators can be associated with pollination shifts. In this clade of understorey herbs, orchid bee pollination is ancestral, and there have been at least ten independent shifts to a hummingbird pollination syndrome. Kay and Grossenbacher (2022) summarized pollinator visitation data across 28 species in Central and South America and found that *Costus* species with bee syndrome traits were occasionally visited by hummingbirds (mean 93.6 % bee visitation, range 72–100 %). In contrast, nearly all species conforming to the hummingbird syndrome were only visited by hummingbirds (mean 99.4 % hummingbird visitation, range 95–100 %). This contrast suggests that the phenotypic range across which hummingbirds make fitness contributions is wider than and overlaps that of bees. Because hummingbirds do not groom pollen the way bees do, they may have the potential to be more efficient at pollen transfer on a per-visit basis, and small increases in visitation could prompt a shift. In what circumstances could visitation be high enough to drive a shift? In other lineages with bee-to-hummingbird shifts, increased relative abundance of hummingbirds at high elevations in the tropics and low elevations in temperate regions are thought to drive shifts (Hamilton and Wessinger, 2022; Dellinger *et al*., 2023). However, in *Costus*, bee- and hummingbird-pollinated species are found at similar elevations and often occur in the same habitats (Kay and Schemske, 2003; Kay and Grossenbacher, 2022). In one *Costus* species that was the focus of extensive observation, Kay and Schemske (2003) found that *C. malortieanus* in Costa Rica was exclusively pollinated by orchid bees during the peak of the wet season, but received substantial additional hummingbird visitation late in the wet season, when other hummingbird floral resources are scarce (Stiles, 1978) (Fig. 10). This work shows that even without a decline in bee visitation or colonization of a new geographic range, changes in the ecological milieu can increase the fitness contribution of a secondary pollinator, potentially providing the opportunity for a shift in pollination syndrome.

‘Modifier traits’ (*sensu* Ohashi *et al*., 2021) that facilitate adaptation to multiple pollinators may also provide a route for pollination shifts. These traits are not well depicted on a simplified 2-D fitness surface because they allow for secondary pollinators on one floral trait axis without incurring trade-offs on another axis. For example, many night-blooming plants stay open the following day and can receive visits from diurnal pollinators if the rewards have not been depleted. In the *Clarkia breweri* example from above, the flowers associated with the hawkmoth syndrome first open in the evening but remain open over the next 2–3 d, and this extended flowering could be considered a modifier trait. The hawkmoths are highly stochastic visitors, both within and across years, and when the flowers are not visited in the evening by hawkmoths, nectar and pollen rewards remain for the suite of less efficient diurnal pollinators, including bees, flies and butterflies, with nectar welling up in the long floral tube to be available to shorter-tongued pollinators (Miller *et al*., 2014; Diaz-Martin *et al*., 2023). Hawkmoth pollination is likely derived from bee pollination in *Clarkia*, and the night/day pattern of floral presentation may mitigate what may otherwise be fitness trade-offs among this wide range of pollinators, leading to broad phenotypic overlap in their fitness contributions. Similarly, in the *Tritoniopsis revoluta* example above, nectar that wells up long floral tubes may be considered a modifier trait because it facilitates effective bee visitation to both long- and short-tubed flowers and flattens the fitness surface (de Merxem *et al*., 2009; Anderson *et al*., 2014; Newman *et al.*, 2025).

### Pollinator shifts may be driven by pollen transfer efficiency and offspring quality

Aside from large decreases in ancestral pollinator abundance, pollinator shifts may occur when a higher-quality pollinator becomes available. Important aspects of pollinator quality, such as pollen transfer efficiency and offspring quality, may be obscured in studies that use visitation rate and/or seed set to assess pollinator effectiveness (e.g. compare Fig. 5A and C). Although ancestral state reconstruction of pollination syndromes has several complexities, especially in highly variable clades, it is likely that many pollination shifts represent transitions away from pollen consumers and local foragers (van der Niet and Johnson, 2012; Rosas-Guerrero *et al*., 2014). For example, hummingbird pollination is typically derived from bee pollination (pollen consumers), with the only evidence for reversals coming from variable clades with high uncertainty in ancestral state reconstruction and/or limited taxon sampling (reviewed in Kay and Grossenbacher, 2022; Barreto *et al*., 2024). Similarly, van der Niet and Johnson (2012) find evidence for frequent shifts from generalized insect pollination to moth pollination in angiosperms, but not the reverse. Additional careful phylogenetic comparative studies of pollination systems could help determine whether this is a general phenomenon.

Non-additive fitness functions (Fig. 5) also help us to understand the adaptive evolution of traits that exclude leaky pollinators and generate more distinct syndromes. When we view pollination shifts retrospectively, the phenotypes we see may reflect both adaptation to the new pollinator and exclusion of the ancestral pollinator to mitigate opportunity trade-offs (Thomson, 2003; Ohashi *et al*., 2021). For example, during a bee-to-hummingbird transition, with both bees and hummingbirds visiting a plant population, the bees could be removing pollen that would otherwise be exported by higher-quality hummingbird pollinators. Our modelling shows that this opportunity trade-off results in non-additive fitness contributions and lower overall fitness for phenotypes that attract both pollinators, and may thus drive selection for filtering traits that exclude the lower-quality pollinator (Fig. 5C). This filtering selection can push phenotypes beyond the optimum expected without the presence of the ancestral pollinator. For example, many traits associated with hummingbird pollination, such as red coloration, dilute nectar and the lack of a landing pad or nectar guides, are thought to primarily be filtering traits against bees (Schemske and Bradshaw, 1999; Castellanos *et al*., 2004). Thus, in hindsight, with shifts between pollinators that differ in quality and cause opportunity trade-offs, we may view very separate adaptive peaks with no obvious ridge between them.

### Hopeful monsters and pollination shifts

One of the exciting finds in the past few decades has been that there are major-effect alleles that contribute to divergence in pollination syndromes (Bradshaw and Schemske, 2003; reviewed in Kay and Sargent, 2009; Hermann *et al*., 2013; Wessinger *et al*., 2023). Major-effect alleles are individual alleles that have a large effect on the phenotypic variation. They have been found for colour, scent and nectar reward, suggesting that these could promote rapid shifts to new pollinators and potentially bypass adaptive valleys between floral forms. It has also been modelled that delayed selfing can both provide reproductive assurance and help to fix major-effect recessive alleles during pollinator shifts, broadening the conditions under which plants can adapt to a higher quality but less reliable pollinator (Wessinger and Kelly, 2018). Indeed, the same study showed that known major-effect alleles involved in shifts from bee to hummingbird pollination are typically recessive and found in self-compatible lineages. Major-effect recessive alleles for phenotypes that attract a new pollinator could be segregating in a population at low frequency, be exposed to selection during a time of pollinator scarcity and/or selfing, and promote adaptation to a new pollinator. On the other hand, most studies of floral morphology have found that many loci of small effect are involved in divergence (Wessinger and Hileman, 2020; Kay and Surget-Groba, 2022). These empirical results suggest that major-effect loci may be more likely to underlie pollinator attraction, and small-effect loci more likely to underlie fit and pollen transfer efficiency, although in most systems the effects of individual traits or loci on pollination have not been decomposed, and this hypothesis needs testing. However, we know that pollination shifts include changes in both attraction and fit, and often comprise multiple aspects of morphology, colour, scent, reward and timing. Thus, substitution of an individual major-effect allele would not bring about a shift on its own, and could end up attracting a new pollinator without fitting it, potentially landing the population in an adaptive valley. Moreover, several of the known major-effect derived alleles associated with hummingbird pollination, such as those conferring red colour, loss of nectar guides and dilute nectar, are thought to function primarily as anti-bee filtering traits (e.g. Schemske and Bradshaw, 1999; Castellanos *et al*., 2004), and in that case would be unlikely to initiate a shift on their own. In summary, the contribution of major-effect alleles does not mean a lineage can bypass a valley in a multivariate adaptive landscape, and the order with which various alleles are likely to fix during a pollination shift remains unknown. Understanding the shape of the adaptive landscape across transitional phenotypes is thus imperative. This could involve dissecting the genetic basis of a pollinator shift followed by functional manipulation of individual genes or phenotypes and testing the resulting phenotypes with both ancestral and derived pollinators. Systems such as monkeyflowers are on the cusp of this type of work (Yuan, 2019).

Reframing pollinator-driven selection with floral adaptive landscapes better encompasses adaptive processes both within and between populations. A less typological view than the MEPP shows that different functional groups of pollinators can often have overlapping fitness contributions across a phenotypic range, especially when considering traits that mitigate phenotypic trade-offs, providing a bridge between what we view as distinct pollination syndromes. Combinations of pollinators that differ in quality, or the overall height of their fitness contribution, can also lead to non-additive fitness contributions that can push populations towards specialization by evolving filtering traits. Better understanding these processes will require more experimental work recreating plausible intermediate trait combinations tested with both ancestral and derived pollinators.

## WHEN AND HOW DOES POLLINATOR-DRIVEN FLORAL DIVERGENCE CONTRIBUTE TO SPECIATION?

The Grant–Stebbins model is fundamentally a model for pollinator-driven speciation, so we now turn to how floral divergence is involved in the origin of new species, and how a framework of floral adaptive landscapes helps us better understand speciation processes. Plant biologists have adopted many different species concepts, and preferences often depend on the context or plant lineage. Nevertheless, in many cases, a variety of concepts apply fairly well to the same taxa and can (but do not always) lead to similar conclusions about species boundaries. Rather than debating the merits of different species concepts, here we address the role of pollinator-driven floral divergence in speciation in light of multiple species concepts: typological, biological, phylogenetic, and ecological or cohesion, while recognizing that these are broad categories comprising multiple named concepts and shades of interpretation.

Under a typological species concept, floral divergence contributes to speciation when the flowers of a population or set of populations are recognized as being relatively invariant but phenotypically distinct from their close relatives. Here, we include morphological and taxonomic species concepts under the broader category of typological. Not all floral divergence would fit this definition; rather, it needs to be more qualitative than quantitative. Qualitative divergence suggests the occupation of separate adaptive peaks (i.e. peaks separated by adaptive valleys) in the floral adaptive landscape, perhaps with those peaks being pushed apart by the evolution of filtering traits because of phenotypic and/or opportunity trade-offs. For example, many studies show that recently diverged sister taxa are associated with shifts between different pollination syndromes that presumably represent separate adaptive peaks (e.g. Manning and Linder, 1992; Johnson *et al*., 1998; Wessinger *et al*., 2016; Lagomarsino *et al*., 2017; Kay and Grossenbacher, 2022). In the *Clarkia* example (Fig. 8), species with overlapping visitor communities can still occupy separate adaptive peaks, and indeed, the taxonomic key for *Clarkia* separates them by diagnostic floral characters (Lewis and Lewis, 1955). Although typological species concepts have been criticized for being subjective and not focused on mechanism, they are the most commonly applied concepts and often align with more mechanistic definitions. As mentioned above, Grant (1949) used the high frequency with which floral characters taxonomically distinguish closely related species to support the importance of floral isolation in speciation. However, even for genera with what he called highly specialized pollination systems, such as bird, bee and long-tongued fly pollination, the majority of taxonomic characters were vegetative, suggesting that pollination is not the sole factor in speciation (Grant, 1949). Similarly, van der Niet and Johnson (2009) systematically reviewed sister species in the Cape Floristic Region and found that species were most often distinguished by a combination of floral and vegetative characters.

Under the biological species concept, floral divergence will contribute to speciation when it promotes interbreeding among individuals with similar floral phenotypes and contributes to reproductive isolation between divergent floral phenotypes. As plant populations adapt to local pollination climates across a geographic range, they will diverge in floral characters in a way that contributes to floral isolation. As stated in Grant and Grant (1965), ‘When the specializations for different classes of pollinators approach or reach a stage of mutual exclusiveness, these differences contribute to the reproductive isolation between the species involved’. Grant (1993*b*, 1994*a*, *b*) further clarified that floral isolation can have both ethological and mechanical components, and that isolation is a by-product of adaptive divergence, rather than being selected for directly. Ethological isolation includes both pollinator preference and floral constancy, whereas mechanical isolation results from differences in the shape and size of flowers that limit pollen transfer because of fit. Others have also noted and studied the indirect effects of floral divergence, especially in style length, on postpollination prezygotic reproductive barriers (reviewed in Yost and Kay, 2009). Floral isolation is generally thought to fit a ‘magic trait’ model, in which the same traits or genes involved in adaptive divergence also cause assortative mating (Servedio *et al*., 2011; Minnaar *et al*., 2019*b*; reviewed in Merrill *et al*., 2024).

Because of the focus on reproductive isolation, the biological species concept is generally applied when populations have at least some geographic overlap (Christie *et al*., 2022). Yet, floral divergence is most likely to occur across geographic space, such that populations are allopatric or parapatric and, importantly, have had the opportunity to diverge in many other ecological niche axes and functional traits. This ecogeographic divergence can preclude young species from coming into contact, leaving floral divergence untested as a reproductive barrier (Sobel, 2014). Mayr, one of the primary architects of the biological species concept, stressed the importance of ecogeographic divergence in driving most speciation, stating ‘all geographical races are also ecological races, and all ecological races are also geographical races’ (Mayr, 1947). Despite much focus in speciation research on mating discrimination and postzygotic barriers (reviewed in Coyne and Orr, 2004), plant biologists have long appreciated the importance of ecogeographic divergence (Turesson, 1922; Clausen *et al*., 1958; reviewed in Sobel *et al*., 2010), perhaps because of the amenability of plants to transplant studies, which are the gold standard method for testing ecogeographic isolation. Many of the classic cases of pollinator-driven speciation are primarily isolated ecogeographically and only show narrow zones of contact, including the Sierra Nevada monkeyflowers and columbines (*Aquilegia*) and the Rocky Mountain *Ipomopsis* (Grant, 1952; Ramsey *et al*., 2003; Aldridge, 2005; Angert and Schemske, 2005). In the *Clarkia* example above, the species only have a narrow point of geographic contact, and niche modelling and a greenhouse reciprocal transplant experiment show strong ecogeographic isolation (Goff *et al*., 2021).

What happens upon secondary contact when populations have experienced divergent selection on floral traits? We see a range of outcomes. Even with clear adaptive divergence to the extent that we recognize distinct pollination syndromes, hybrid zones and clines are often formed upon secondary contact. For example, the classic *Aquilegia* species pair studied by Grant in the Sierra Nevada of California is primarily allopatric, with *A. formosa* growing along streams at low to mid-elevation and *A. pubescens* growing in open rocky habitat in the high alpine. The former exhibits a hummingbird pollination syndrome and the latter a hawkmoth pollination syndrome, yet they readily form hybrid zones when rocky scree fields spill across high-elevation streams where their ranges meet (Grant, 1952, 1993*a*; Noutsos *et al*., 2014). These hybrid zones form even though their floral divergence contributes to reproductive isolation through assortative mating based on both pollinator preference and morphological fit (Hodges and Arnold, 1994; Fulton and Hodges, 1999). In *Ipomopsis aggregata* and *I. tenuituba*, differences in pollinator behaviour among contact zones lead to different hybridization outcomes, showing that the strength of floral reproductive isolation can be context-dependent (Aldridge, 2005; Aldridge and Campbell, 2007; Bischoff *et al*., 2015).

In other cases, young species experience very strong floral isolation in regions of sympatry. Strong isolation may be more likely with distant adaptive peaks, especially those in which filtering traits evolve in response to phenotypic and opportunity trade-offs. For example, differences in orchid bee versus hummingbird pollination syndromes between sympatric spiral ginger species confer nearly complete ethological isolation and hybrids are rare, although typically there is limited sympatry between sister species (Kay and Schemske, 2003; Vargas *et al*., 2020). Similar patterns are seen in bee- versus hummingbird-pollinated monkeyflowers and *Penstemon*, with little observed hybridization occurring in areas of sympatric overlap (Chari and Wilson, 2001; Ramsey *et al*., 2003). However, strong sympatric reproductive isolation can even occur when recently diverged forms have overlapping pollinators but different effects on pollinator behaviour and mechanical fit (e.g. Anderson *et al*., 2016; Minnaar *et al*., 2019*b*).

When there is no natural zone of sympatry, experiments can be used to quantify floral isolation under the biological species concept. For example, reciprocal translocations of the geographically isolated sister *Clarkia* species (Fig. 8) show they experience very similar visitation when experimentally brought together at sites within each of their ranges, suggesting that the difference in visitors is largely due to habitat rather than floral traits, although pollen transfer is reduced because of the morphological differences (Miller *et al*., 2014; Kay *et al*., 2019). Similarly, Newman *et al.* (2015) reciprocally translocated floral ecotypes of *Nerine humilis* and found no evidence of ethological isolation by different pollinator assemblages but substantial local adaptation in traits affecting fit and pollen transfer, which would likely contribute to mechanical isolation. These studies suggest that, for allopatric taxa, floral isolation cannot simply be assumed from floral traits or visitors alone, and should be tested. Moreover, partial floral isolation could result in reinforcing selection upon sympatric contact when hybrids are less fit (e.g. Hopkins and Rausher, 2012), yet it is unknown how common or important reinforcement is for floral isolation.

In many cases, floral divergence contributes to reproductive isolation, but is rarely, if ever, the sole barrier to gene flow (Kay and Sargent, 2009). Rather, ecogeographic isolation is more likely to initiate biological speciation, with pollinators playing a potential role in divergence of some niche axes and a supporting role in generating reproductive isolation in regions of sympatric contact. An advantage of the biological species concept framework is that the strength of floral isolation and other forms of reproductive isolation can be directly compared on the same linear scale (Sobel and Chen, 2014). Indeed, in a review of reproductive isolating barriers in plants, ecogeographic isolation, defined as geographic isolation resulting from intrinsic biological differences between taxa, and immigrant inviability, both of which prevent sympatric mating opportunities, were some of the strongest reported barriers, although floral isolation was also strong (Christie *et al*., 2022). Nevertheless, researchers may be biased towards choosing study systems in which floral isolation and pollination are thought to be important *a priori*, and the relative importance of floral isolation to total reproductive isolation is still unknown.

Is floral divergence sufficient to prevent gene flow and introgression? This question bears on whether pollinator-driven divergence results in speciation under the phylogenetic species concept, which places primary importance on phylogenetic independence or reciprocal monophyly. Recent work in *Penstemon* shows that only a small number of unlinked genetic loci distinguish species with different pollination syndromes and that most genetic variation is shared across species (Wessinger *et al*., 2023). Similarly, recent phylogenomic and genetic investigation of bee- versus hummingbird-pollinated monkeyflowers with strong ecogeographic and floral isolation provides evidence for historical introgression between species (Nelson *et al*., 2021). Another study of pollination ecotypes in South African *Erica* finds similar results (Musker *et al*., 2024). Thus, even with few obvious hybrids, floral isolation may seldom be strong enough to completely prevent gene flow. These studies also highlight the importance of natural selection in maintaining species differences in the face of gene flow. In contrast, phylogenomic and population genetic investigation of the spiral gingers shows very limited introgression among species, despite extensive sympatric coexistence and broad interfertility across the genus (Kay and Schemske, 2008; Surget-Groba and Kay, 2013; Uckele *et al*., 2024). Furthermore, population genetic studies of *Clarkia* (Fig. 8) show strong genetic isolation despite their interfertility even at the narrow point of geographic contact (Diaz-Martin *et al*., 2023). These studies suggest that when incipient species with divergent pollination systems experience sympatric contact, they only sometimes meet the criteria for the phylogenetic species concept, and the outcome can depend on how stringently this concept is applied and which parts of the genome are considered. Because of how divergent floral adaptation can happen in a portion of an ancestral species’ range, we expect that floral ecotypes may not always be reciprocally monophyletic, but rather may form progenitor-derivative species pairs, similar to what is seen for edaphic adaptation (e.g. Baldwin, 2005).

Ecological and cohesion concepts focus less on the boundary between species than the cohesive forces within species, such as stabilizing selection, ecological niche conservatism, and gene flow among populations (Van Valen, 1976; Templeton, 1998). We note that the biological species concept also identifies the importance of interbreeding among populations of the same species (Mayr, 1947). With this view of species, pollinator-driven floral divergence may be more important as a cohesive force among ecologically similar populations than an isolating force between incipient species. Since the pollination climate is likely to co-vary with the environment, populations experiencing similar selective pressures from the abiotic environment and non-pollinator aspects of the biotic environment are likely to have similar flowers that promote gene flow and maintain the ecological cohesion of the species. *Aquilegia* may provide an insightful example. Hummingbirds in California may be limited to low- and middle-elevation riparian habitats where they can successfully breed during the temperate summer (Grant and Grant, 1968; Hamilton and Wessinger, 2022). Hummingbird pollination of *A. formosa* provides cohesive gene flow among the plants living in these habitats. In contrast, hawkmoths are able to migrate seasonally into the alpine and provide cohesive gene flow among similarly adapted *A. pubescens* plants (Noutsos *et al*., 2014). In this case, pollinators contribute to speciation regardless of whether there is floral isolation upon sympatric contact.

Regardless of species concept, we see commonalities relating to the role of pollinator-driven divergence. Notably, floral divergence alone is rarely sufficient to define species, and typically evolves with other ecological divergence across a geographic range. Similarly, in addition to other reproductive barriers and cohesive forces, floral divergence may contribute to reproductive isolation between incipient species and promote cohesion among populations under stabilizing selection and with similar ecological niches. Grant (1949) himself indicated that floral isolation may play a secondary role in speciation: ‘Ethological isolation may operate to reinforce an isolation originally set up by geographical and ecological factors.’ While this does not mean that pollinators are not important in driving parts of the speciation process, it does mean that they are seldom the sole drivers of the process and should not be considered in isolation. Adopting a framework of floral adaptive landscapes and investigating how they vary, correlate with other aspects of the environment and contribute to floral isolation across the geographic landscape will facilitate a unified view of geographic, ecological and floral divergence in plant speciation.

## FUTURE DIRECTIONS

Compared with the Grant–Stebbins model, a floral adaptive landscape framework offers a more nuanced and realistic approach to understanding floral adaptation and can better explain floral divergence and its contributions to plant speciation. Rather than adapting to the most effective pollinator, plants adapt to maximize fitness (i.e. flowers are adaptive responses to the peaks and troughs of fitness landscapes and the combined effects of all floral visitors within a community). Although quantifying adaptive landscapes across floral visitors, phenotypes and geographic sites is a daunting empirical task, we have attempted to highlight examples and opportunities throughout this review that move the field towards that ideal. The key will be first adjusting our framing and approach. Then we can assess which questions can be addressed with existing data but need a fresh lens, and which questions will require new data. For example, studies framed from an MEPP perspective may already provide unemphasized data on when and by how much less effective pollinators contribute to fitness or selection on floral traits. In contrast, we propose expanding data collection in certain areas, including selection through male function, variation in offspring quality among visitors, geographic variation in pollinator-driven selection, and the interplay between ecogeographic and floral divergence in speciation. The first two can be accomplished more easily with current technology, while the latter may require age-old reciprocal transplant techniques spanning vegetative and reproductive life stages. We see promise in modelling approaches to complement empirical studies, especially for complicated issues like non-additive combinations of visitor-specific fitness contributions. We expect that moving away from the MEPP and adopting a floral adaptive landscape perspective will provide transformative insight into processes of floral adaptation, ecotypic divergence, and speciation.
